# Sequential [^18^F]FDG-[^18^F]FMISO PET and Multiparametric MRI at 3T for Insights into Breast Cancer Heterogeneity and Correlation with Patient Outcomes: First Clinical Experience

**DOI:** 10.1155/2019/1307247

**Published:** 2019-01-08

**Authors:** Piotr Andrzejewski, Georg Wengert, Thomas H. Helbich, Heinrich Magometschnigg, Dietmar Georg, Marcus Hacker, Pascal Baltzer, Paola Clauser, Panagiotis Kapetas, Petra Georg, Wolfgang Wadsak, Katja Pinker

**Affiliations:** ^1^Department of Radiation Oncology, Medical University of Vienna, Austria; ^2^Christian Doppler Laboratory for Medical Radiation Research for Radiation Oncology, Medical University Vienna, Austria; ^3^Department of Biomedical Imaging and Image-guided Therapy, Molecular and Gender Imaging Medical University of Vienna, Austria; ^4^Department of Biomedical Imaging and Image-guided Therapy, Division of Nuclear Medicine, Medical University of Vienna, Austria; ^5^Department of Radiology, Breast Imaging Service, Memorial Sloan Kettering Cancer Center, New York, NY, USA

## Abstract

The aim of this study was to assess whether sequential multiparametric ^18^[F]fluoro-desoxy-glucose (^18^[F]FDG)/[^18^F]fluoromisonidazole ([^18^F]FMISO) PET-MRI in breast cancer patients is possible, facilitates information on tumor heterogeneity, and correlates with prognostic indicators. In this pilot study, IRB-approved, prospective study, nine patients with ten suspicious breast lesions (BIRADS 5) and subsequent breast cancer diagnosis underwent sequential combined [^18^F]FDG/[18F]FMISO PET-MRI. [^18^F]FDG was used to assess increased glycolysis, while [^18^F]FMISO was used to detect tumor hypoxia. MRI protocol included dynamic breast contrast-enhanced MRI (DCE-MRI) and diffusion-weighted imaging (DWI). Qualitative and quantitative multiparametric imaging findings were compared with pathological features (grading, proliferation, and receptor status) and clinical endpoints (recurrence/metastases and disease-specific death) using multiple correlation analysis. Histopathology was the standard of reference. There were several intermediate to strong correlations identified between quantitative bioimaging markers, histopathologic tumor characteristics, and clinical endpoints. Based on correlation analysis, multiparametric criteria provided independent information. The prognostic indicators proliferation rate, death, and presence/development of recurrence/metastasis correlated positively, whereas the prognostic indicator estrogen receptor status correlated negatively with PET parameters. The strongest correlations were found between disease-specific death and [^18^F]FDG_mean_ (*R*=0.83, *p* < 0.01) and between the presence/development of metastasis and [^18^F]FDG_max_ (*R*=0.79, *p* < 0.01), respectively. This pilot study indicates that multiparametric [^18^F]FDG/[^18^F]FMISO PET-MRI might provide complementary quantitative prognostic information on breast tumors including clinical endpoints and thus might be used to tailor treatment for precision medicine in breast cancer.

## 1. Introduction

Breast cancer is a complex disease with remarkable intratumoral heterogeneity resulting in different clinical and phenotypic presentations, treatment responses, and outcomes [1–4]. Magnetic resonance imaging (MRI) and positron emission tomography (PET) play an important role in staging, therapy monitoring, and follow-up of breast cancer [5]. Recently, hybrid PET/MRI scanners have become clinically available. An inherent advantage of PET-MRI is that it provides noninvasive qualitative and quantitative in vivo information as well as spatiolongitudinal monitoring of the tumor microenvironment and its interaction, while invasive tissue sampling provides just “snapshots” of specific tumor regions.

The routinely used radiotracer [^18^F]FDG depicts increased tissue glycolysis indicative of malignancy and has high sensitivity for the detection of breast cancer. Initial studies have already demonstrated the potential of 2-deoxy-2-[^18^F]fluoro-D-glucose ([^18^F]FDG) PET/MRI for breast cancer diagnosis, characterization, and prognosis [6, 7]. However, for PET imaging, several other radiotracers that specifically target cancer hallmark processes relevant for cancer development, progression, and treatment resistance have also been developed, such as [^18^F]fluoromisonidazole ([^18^F]FMISO) for imaging hypoxia [8], [^18^F]fluorothymidine ([^18^F]FLT) for imaging proliferation [9], and [^18^F]fluoroestradiol ([^18^F]FES) for imaging hormone receptor status. The combined use of different radiotracers may add diagnostic, predictive, and prognostic information, and therefore, the full potential of PET-MRI in breast cancer is yet to be realized.

In breast cancer, tumor hypoxia has been recognized as an important feature and a key driver of intratumoral heterogeneity which in turn leads to the development of cell clones with an aggressive and treatment-resistant phenotype characterized by rapid progression and a poor prognosis [10–12]. Hypoxia has been implicated as a confounder of the efficacy of cancer therapies as well as a prognostic factor for disease progression, metastases, and survival [10–12]. PET imaging using [^18^F]FMISO has been shown to identify hypoxic tumor subvolumes and track spatiotemporal dynamics. Therefore, it might provide additional information to [^18^F]FDG particularly on breast cancer heterogeneity and have additional prognostic value. Therefore, the aim of this pilot study was twofold:

- To assess whether combined sequential, dual tracer multiparametric PET-MRI at 3T in primary breast cancer patients is possible and facilitates information on tumor heterogeneity
- To investigate associations with recurrence and disease-specific patient survival

To reach this goal, we used multiple MRI parameters (high-resolution T2-weighted, dynamic contrast-enhanced (DCE)-MRI, and diffusion-weighted imaging (DWI) with apparent diffusion coefficient (ADC) mapping) and combined these parameters with that from PET using the radiotracers [^18^F]FDG for the assessment of glycolytic metabolic activity and [^18^F]FMISO for the detection of tumor hypoxia.

## 2. Materials and Methods

The Institutional Review Board approved this prospective, single-institution study, and all patients gave written, informed consent.

### 2.1. Patients

Nine patients (mean age of 53.2 years) with 10 breast cancer lesions who fulfilled the following inclusion criteria were included: 18 years or older; not pregnant; not breastfeeding; imaging finding at mammography or breast ultrasonography highly suggestive of malignancy (BI-RADS 5); no previous treatment; and no contraindications for MRI or MRI contrast agents (1). For all patients, the following information was recorded: age, histologic type, tumor grade, receptor status, tumor proliferation rate (Ki67), nodal status, presence of regional or distant metastases, date of progression (local recurrence, distant metastases) to determine duration (months) of recurrence-free survival (RFS), and date and cause of death or date of last follow-up to determine duration (months) of disease-specific death (DSS).

### 2.2. Imaging

All patients underwent sequential combined multiparametric dual tracer [^18^F]FDG/[^18^F]FMISO PET-MRI at 3T with examinations performed no more than 7 days apart.

### 2.3. MR Imaging

The MRI examinations were performed with the patient in the prone position using a 3T MRI (Tim Trio, Siemens, Erlangen, Germany) and a four-channel breast coil (InVivo, Orlando, FL, USA). The MRI protocol included the following sequences:

1. T2-weighted with fat suppression: axial turbo inversion recovery magnitude (TIRM) sequence: repetition time/echo time (TR/TE) 4800/59 msec; field of view (FOV) 340 mm; 44 slices at SI 4 mm; flip angle (FA) 120°; matrix 384 × 512; acquisition time (AT): 2 min 35 sec
2. T2-weighted with fat suppression: turbo spin echo sequence: TR/TE = 4630/194 msec; FOV 340 mm; 65 slices at slice thickness (SI) 2.5 mm; FA 128°; matrix 384 × 640; AT: 2 min 48 sec
3. DWI: axial three-acquisition trace diffusion-weighted, double-refocused, single-shot echo-planar imaging sequence with inversion recovery fat suppression: TR/TE/time of inversion (TI) 8000/59/210 msec; FOV 360 × 202 mm; 24 slices at 5 mm; matrix 172 × 96 (50% oversampling); b-values 50 and 850 s/mm, AT: 2 min 56 sec
4. DCE-MRI: transversal T1-weighted time-resolved angiography with stochastic trajectories (TWIST) with water excitation fat saturation: TR/TE 6.23/2.95 ms; FA 15°, FOV 196 × 330 mm 144 slices; spatial resolution 0.9 × 0.9 × 1 mm; temporal interpolation factor 2; temporal resolution 14 s; matrix 384 × 384; one average; center k-space region with a resampling rate of 23%; reacquisition density of peripheral k-space 20%; AT: 6 min 49 sec

A standard dose (0.1 mmol/kg of bodyweight) of Gd-DOTA Dotarem was administered intravenously as a bolus with a power injector (Spectris Solaris EP; Medrad) at 4 mL/s followed by a saline flush.

### 2.4. PET Imaging

All PET studies were acquired using a hybrid PET/CT (computed tomography) (Biograph 64 TruePoint PET/CT system, Siemens, Erlangen/Germany). For [^18^F]FDG PET/CT, patients fasted for five hours. Patients were injected with 3 MBq [^18^F]FDG and [^18^F]FMISO per kilogram bodyweight on different days. Scanning was started after an uptake time of 45 min for [^18^F]FDG and 210–240 min for [^18^F]FMISO. For both radiotracers, a prone PET dataset with low-dose unenhanced CT scans was recorded for attenuation correction. The same imaging and postreconstruction parameters were used for both PET/CT studies. Images were reconstructed using the iterative TrueX algorithm, which incorporates a specific correction for the point-spread function in addition to commonly used correction factors [13, 14]. Four iterations per 21 subsets were used, with a matrix size of 168 × 168, a transaxial FOV of 605 mm (pixel size 3.6 mm), and a section thickness of 5 mm. Further technical details are provided by the manufacturer [15]. For both examinations, patient positioning and the scan time took on average ∼40 minutes.

### 2.5. Image Fusion

**Figure 1:**
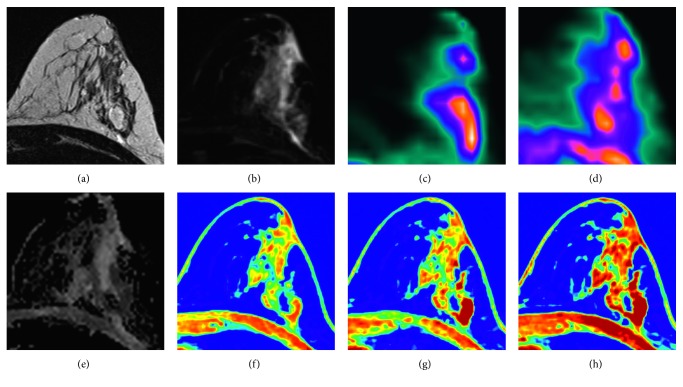
46-year-old patient with hormone receptor positive/Her2 negative invasive ductal carcinoma in the left breast. Multiparametric [^18^F]FDG/[^18^F]FMISO PET-MRI : cancer presented as a segmental heterogenous persistently enhancing nonmass enhancement. (f–h) DCE_native_, DCE_early_, and DCE_delayed_ with perifocal edema. (a) T2-weighted without fat saturation. (b) T2-weighted with fat saturation. The lesion showed moderate uptake of (c) [^18^F]FDG (SUV_max_ = 6.5) and (d) [^18^F]FMISO (SUV_max_ = 1.3) with restricted diffusion visualized on the ADC map (e).

All images were coregistered and fused to allow delineation of regions of interest (ROIs). An automatic, region-based, rigid, fine image registration protocol was used in the Mirada RTx software (Mirada Medical Ltd.) and followed with manual adjustments if necessary (5–10 min per pair of images). The MRI-PET registration was performed in two steps: first, the T2-weighted MRI and the corresponding CT were registered, and second, that transformation was applied to the PET dataset (Figure S1). Within the MRI series, the initial frame of reference was used as the starting point for rigid registration, correcting for possible patient movement. At the end of the process, all images were aligned with the T2-weighted MRI (Figure 1). For the purpose of voxel-by-voxel analysis, all datasets were also resampled to the resolution of the PET: 4.1 × 4.1 × 3.0 mm.

### 2.6. Data Analysis

For each patient, the tumor was delineated by an experienced breast radiologist and nuclear medicine physician, and the following parameters were derived from the delineated regions of interest (ROI): volume (cm^3^); mean signal intensity on T2-weighted images with and without fat saturation as well as on native, early, and delayed DCE-MR images (DCE_native_, DCE_early_, and DCE_delayed_, respectively); initial enhancement (IE) ratio and washout (WO) ratio (IE ratio defined as (DCE_early_−DCE_native_)/DCE_native_ and WO ratio defined as (DCE_delayed_−DCE_early_)/DCE_native_)); mean ADC value on DWI; mean and maximum standard uptake value (SUV) for each tracer; and tumor to background (TBR) ratio (calculated as the ROI SUV normalized to the SUV measured in the patients' aorta) for each tracer. For all PET-derived parameters, a ratio between [^18^F]FDG and respective [^18^F]FMISO parameter was calculated.

### 2.7. Statistical Analysis

Imaging findings and calculated ratios were compared with clinical data (i.e., histopathologic tumor characteristics including hormone receptor status (estrogen, progesterone and human epidermal growth factor receptor 2 receptor) and proliferation rate (Ki67)) and patient outcomes (i.e., disease-specific death (DSS) and presence/development of metastases) using multiple correlation analysis.

Person correlation coefficients (R) were also calculated for each parameter pair. Statistically significant correlations were color-coded (blue for negative correlations and green for positive correlations). *p* values were marked in green for significance at the ≦0.05 level (2-tailed) and red for that at the ≦0.01 level. For image-derived parameters, Person correlation coefficients were additionally calculated on a voxel-by-voxel level. For PET-derived TBR, a value in each voxel was considered in relation to the SUV_aorta_; hence, TBR_mean_ and TBR_max_ were not used [16–18]. Voxel-by-voxel analysis was additionally performed for [^18^F]FMISO_TBR_ > 1.4 and [^18^F]FDG_TBR_ > 2.0 parameters, where only the voxels in which TBR was larger than 1.4 or 2.0, respectively, were considered, and only moderate and strong correlations were color-coded (Figure S2).

## 3. Results

Relevant clinical data, pathological tumor characteristics, and clinical endpoints for each patient are summarized in Table 1. The mean follow-up time for all patients was 31.6 months (range, 3–60 months). One patient presented with one breast cancer in each breast which were of different tumor biology; we therefore considered this patient as two separate cases (ID 6: IDC ER/PR negative, Her2 positive, and Ki67 90%; ID 7: IDC ER/PR positive, Her2 negative, and Ki67 20%). There were six (60%) cases of distant metastases present at diagnosis, two (20%) cases of distant metastases present at six and 17 months, respectively, and two (20%) cases of no recurrence. In five cases (50%), the patient died of breast cancer during the follow-up period at a median interval of 10 months (range, 3–19 months).

**Figure 2:**
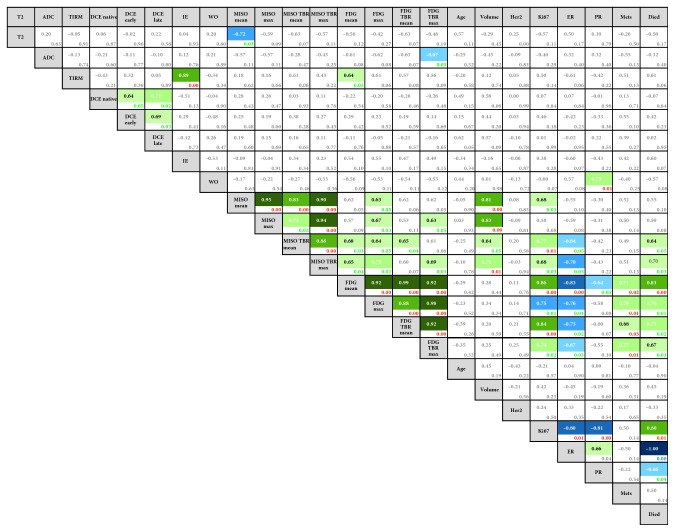
Graph showing numeric and color-coded Person's correlation coefficients of all imaging and clinical parameters. Statistically significant correlations are color-coded (blue for negative correlations and green for positive correlations). *p* values are presented in the bottom right corner of each cell (green for significance at the 0.05 level (2-tailed) and red for that at the 0.01 level). The statistically significant correlations ranged from moderate to strong. Abbreviations: T2w, T2-weighted magnetic resonance imaging (MRI); ADC, apparent diffusion coefficient; TIRM, turbo inversion recovery magnitude MRI; DCE, dynamic contrast enhanced MRI; IE, initial enhancement ratio; WO, washout ratio; FMISO/FDG_mean_, region of interest mean standard uptake value (SUV) of [^18^F]fluoromisonidazole or ^18^[F]fluoro-deoxy-glucose; FMISO/FDG_max_, region of interest maximum SUV of FMISO or FDG; TBR_mean_/_max_, tumor to background ratio based on mean/max SUV in the tumor normalized to mean SUV in the aorta; Ki67, proliferation rate; Her2, human epidermal growth factor receptor 2; ER, estrogen receptor; PR, progesterone receptor; Mets, metastasis.

Several intermediate to strong correlations were identified between quantitative imaging markers, histopathologic tumor characteristics, and outcome measures as summarized in Figure 1. Signal intensity on T2-weighted images and ADC_mean_ values correlated negatively with [^18^F]FMISO and [^18^F]FDG parameters; the strongest correlations were found between signal intensity on T2-weighted images and [^18^F]FMISO_mean_ (*R*=−0.72, *p*=0.03) and between ADC_mean_ and [^18^F]FDG_TBRmax_ (*R*=−0.67, *p*=0.05). Among the quantitative MR imaging parameters, the strongest correlation was found between signal intensity on T2-weighted fat-saturated images and IE ratio (*R*=0.89, *p* < 0.01). Among the PET parameters, a noticeable trend of moderate correlation between the PET markers was observed in the middle cluster of the graph in Figure 2. A moderate-to-strong positive correlation was found between [^18^F]FMISO_max_ and lesion volume (*R*=0.83, *p* < 0.01) and between [^18^F]FDG_TBRmax_ and [^18^F]FMISO_TBRmax_ (*R*=0.69, *p*=0.03).

Both [^18^F]FMISO_TBRmean_ (*R*=0.77, *p* < 0.01) and [^18^F]FDG_mean_ (*R*=0.86, *p* < 0.01) correlated strongly with Ki67. There was a negative correlation of PET parameters with ER; the strongest correlation was found between [^18^F]FDG_mean_ and ER (*R*=0.83, *p* < 0.01) (Table 2).

Clinical endpoints such as death and presence/development of metastasis correlated positively with PET parameters. The strongest correlation was found between DSS and [^18^F]FDG_mean_ (*R*=0.83, *p* < 0.01) and between metastases and [^18^F]FDG_max_ (*R*=0.79, *p* < 0.01). There were also moderate correlations for DSS with both [^18^F]FMISO_TBRmean_ and [^18^F]FMISO_TBRmax_. The calculated ratio of [^18^F]FDG_TBRmax_/[^18^F]FMISO_TBRmax_ had a strong correlation with the presence/development of metastases (*R*=0.69, *p*=0.03).

In voxel-by-voxel analysis, no significant correlations were identified between MRI parameters or MRI and PET parameters. Only a weak correlation between two PET tracers without restricted TBR was found (Figure S2).

## 4. Discussion

Results of high-throughput molecular profiling studies have revealed that breast cancer is a disease with a remarkable tumor heterogeneity resulting in varying genetic, phenotypic, and behavioral characteristics; clinical presentations; and treatment responses [1, 4–9]. This recognized tumor heterogeneity, and the lack of understanding thereof significantly contributes to treatment failures and breast cancer deaths. Breast cancer heterogeneity is driven by tumor genomic instability and selective pressures from the tumor microenvironment with hypoxia being one of the most significant factors. Tumor hypoxia leads to the development of cell clones with an aggressive and treatment-resistant phenotype characterized by rapid progression and a poor prognosis [10–12]. Hypoxia has been implicated as a confounder of the efficacy of cancer therapies as well as a prognostic factor for disease progression, metastases, and survival [10–12]. To date, current invasive tools and conventional imaging technologies cannot provide a comprehensive assessment of breast cancer heterogeneity of the whole tumor. In this context, there is a unique opportunity for noninvasive functional imaging with PET-MRI in breast cancer using multiple MRI parameters, yet to date its potential has not been explored in detail. To our knowledge, this is the first study that investigates whether combined sequential, dual tracer multiparametric PET-MRI at 3T in primary breast cancer patients is possible, facilitates information on tumor heterogeneity, and correlates with survival outcomes. In this pilot study performed in a limited number of patients, we show that a comprehensive noninvasive assessment of breast cancer biology, heterogeneity, hypoxic tumor microenvironment, and the degree to which breast cancer is successful in adapting to sustain growth is feasible with in vivo functional imaging.

We demonstrated the feasibility and potential of multiparametric [^18^F]FDG/[^18^F]FMISO PET-MRI in breast cancer patients. Our pilot study showed several intermediate to strong correlations between quantitative imaging biomarkers, histopathologic tumor characteristics, and clinical endpoints. Based on correlation analysis, multiparametric criteria provided independent information on tumor heterogeneity that is known to significantly contribute to treatment failures and breast cancer deaths; in the future, this information can be used to personalize cancer treatment.

Multiparametric MRI of the breast is already the state-of-the-art for the staging of breast cancer patients, and hybrid PET/MR scanners are now clinically available; therefore, this information will readily be available. In this pilot study, there was no correlation of MRI parameters and clinical endpoints, which might be due to the small sample size as prior studies have already demonstrated both a prognostic and predictive potential [19–26]. For instance, Durando et al. [25] showed that ADC can be a prognostic factor for malignancy aggressiveness; Dietzel et al. [20] and Alduk et al. [19] showed that MRI-derived parameters associate with nodal status, predict positive axillary lymph nodes, or larger tumor sizes; and Bae et al. [23] found significant association between pretreatment T2 MRI features with recurrence-free survival. On the contrary, [^18^F]FDG and [^18^F]FMISO PET parameters showed a moderate-to-strong positive correlation with tumor proliferation rate (Ki67) (a poor prognostic indicator [27]) and DSS. [^18^F]FDG demonstrated strong positive correlation with the presence/development of metastasis as well as a strong negative correlation with ER positivity (a good prognostic indicator [28]). The results of the current study support findings by others that have demonstrated that [^18^F]FDG and MRI parameters may aid in the assessment of tumor aggressiveness and metastatic potential, which further highlights the value of noninvasive functional imaging for improved risk assessment [7].

Although, in this pilot study, patients with high [^18^F]FMISO uptake did not show the tendency to present with or develop metastasis, there was a positive correlation yet weaker correlation than [^18^F]FDG_mean_ of the [^18^F]FDG_TBRmax_/[^18^F]FMISO_TBRmax_ ratio with presence of metastasis. These interesting findings suggest that whenever within a metabolically active tumor there is significant hypoxia, which is a key driver for the emergence of treatment resistant and aggressive clones, these patients are at an increased risk of metastases and might benefit from intensified treatment or tighter follow-up. In addition, there was a correlation of increased [^18^F]FMISO uptake with death, which further suggests that these tumors are more treatment-resistant. This is in agreement with findings from Asano et al. who demonstrated that higher [^18^F] FMISO_TBR_ and ER negativity were independent predictors of shorter disease-free survival, where higher [^18^F] FMISO_TBR_ was associated with higher plasma levels of angiogenic hypoxic markers [29]. Cheng et al. also showed that [^18^F]FMISO PET/CT can be used to predict primary endocrine resistance in ER-positive breast cancer [8].

Voxel-by-voxel analysis showed only low correlations between two PET tracers, whereas descriptive statistics suggested higher correlations. This implies that tumors characterized by regions of increased metabolism have at the same time highly hypoxic subregions that are spatially disjointed. It has to be noted that the voxel-wise analysis is per se prone to spatial inaccuracies (i.e., dependent on image registration and temporal difference between the PET scans). Despite the relatively low resolution of the PET scans (resulting from the physics of the annihilation process) combined with the resampling of the image dataset, the voxel-by-voxel analysis error might be locally exceeding the dimension of one voxel. Therefore, our findings will have to be further validated, preferentially using a simultaneous hybrid PET/MRI.

The main limitation of this pilot study, by virtue of the novelty of the imaging protocol, is the small patient cohort, limiting statistical analysis, and therefore, no strong conclusions can be drawn. In addition, due to this fact, we did not perform modeling to assess which parameters from [^18^F]FDG/[^18^F]FMISO PET-MRI are most strongly associated with RFS and DSS. Further larger studies, preferably with simultaneous hybrid PET/MRI scanners, are necessary to confirm these findings and to clarify which parameters independently or jointly provide prognostic information on breast tumors, including clinical endpoints. In this study, we performed both qualitative and quantitative analysis of [^18^F]FDG/[^18^F]FMISO PET-MRI data. The T2w MRI signal intensity was not normalized, and as it can vary from scan to scan depending on the gain settings, it cannot be considered quantitative.

## 5. Conclusions

In conclusion, according to these preliminary results, multiparametric [^18^F]FDG/[^18^F]FMISO PET-MRI provides complementary quantitative prognostic information on breast tumors including clinical endpoints and thus might be used to tailor treatment for precision medicine in breast cancer.

## Figures and Tables

**Table 1:** Summary of clinical data, immunohistochemical (IHC) status, and molecular pathology derived via IHC and clinical endpoints for all patients.

ID	Age	Histo	Grade	ER	PR	Her2	Ki67 (%)	Disease-specific death	Metastases
1	55	IDC	3	Negative	Negative	Negative	70	Y	Y
2	32	IDC	3	Negative	Negative	Negative	70	Y	Y
3	46	IDC	2	Positive	Positive	Negative	20	N	Y
4	68	IDC	3	Negative	Negative	Negative	90	Y	Y
5	36	IDC	3	Positive	Negative	Negative	80	N	Y
6	54	IDC	3	Negative	Negative	Positive	90	Y	Y
7	54	IDC	3	Positive	Positive	Negative	20	Y	Y
8	68	IDC	3	Positive	Negative	Negative	30	N	N
9	44	IDC	2	Positive	Positive	Negative	30	N	N
10	75	IDC	3	Negative	Negative	Negative	90	N	Y

ER, estrogen receptor; PR, progesterone receptor; Her2, human epidermal growth factor receptor 2; Histo, histologic subtype; Ki67, proliferation rate; IDC, invasive ductal carcinoma.

**Table 2:** Imaging data of all study cases.

ID	Volume	TIRM	T2w	ADC	DCE_native_	DCE_early_	DCE_delayed_	IE	WO	FMISO_mean_	FMISO_max_	FMISO TBR_mean_	FMISO TBR_max_	FDG_mean_	FDG_max_	FDG TBR_mean_	FDG TBR_max_
1	35.5	379	348	1271	141	697	709	3.9	0.1	0.8	1.3	1	1.6	6.3	16.5	2.7	7.2
2	29.5	251	176	n/a	74	250	205	2.4	−0.6	1.4	3.7	0.9	2.5	7.4	24.1	3.7	12.1
3	15	164	273	1242	406	713	756	0.8	0.1	0.8	1.3	0.6	0.9	2.3	6.5	1.2	3.3
4	193.9	199	216	1208	525	813	951	0.5	0.3	1.8	4.3	1.1	2.7	5.1	16.9	2.1	7
5	16.3	229	n/a	1056	275	503	534	0.8	0.1	1.3	2.1	1	1.6	5.7	16.2	3.2	9
6	10	206	168	955	244	511	539	1.1	0.1	1.2	2	0.8	1.3	8.1	17.8	4.1	8.9
7	91	197	344	892	262	264	793	0	2	1.2	2.7	0.7	1.5	3.5	13.5	1.7	6.4
8	8.5	136	416	1182	329	340	435	0	0.3	0.5	0.9	0.4	0.7	1.1	3.2	0.5	1.3
9	12.5	171	176	1509	155	163	442	0.1	1.8	1	1.5	0.8	1.3	1.7	2.5	1	1.5
10	176	256	139	685	350	600	604	0.7	0	2	4.4	1.3	2.8	7.6	17.3	3.8	8.7

T2w, intensity on T2-weighted magnetic resonance imaging (MRI) (arb. unit); ADC, apparent diffusion coefficient (×10^−6^ mm^2^/s); TIRM, intensity on turbo inversion recovery magnitude MRI (arb. unit); DCE, intensity on dynamic contrast enhanced MRI (arb. unit); IE, initial enhancement ratio (arb. unit); WO, washout ratio (arb. unit); FMISO/FDG_mean_, region of interest mean standard uptake value (SUV) of [^18^F]fluoromisonidazole or [^18^F]fluoro-deoxy-glucose (g/ml); FMISO/FDG_max_, region of interest maximum SUV of FMISO or FDG (g/ml); TBR_mean/max_, tumor to background ratio based on mean/max SUV in the tumor normalized to mean SUV in the aorta (arb. unit).

## Data Availability

The imaging data used to support the findings of this study are available from the corresponding author upon request.

## Supplementary Materials

Supplementary Materials Figure S1: example of the registration of the CT_FDG_, CT_FMISO_, and T2w MRI: (A) CT_FDG_; (B) T2w MRI; (C) fusion of registered A and B; (D) CT_FDG_; (E) CT_FMISO_; (F) fusion of registered D and E. Figure S2: graph showing numeric and color-coded Person's correlation coefficients of all imaging parameters performed voxel-by-voxel within each patient's delineated ROIs. Abbreviations: T2w, T2-weighted magnetic resonance imaging (MRI); TIRM, turbo inversion recovery magnitude MRI; ADC, apparent diffusion coefficient; IE, initial enhancement ratio; WO washout ratio; FMISO/FDG_TBR_, tumor to background ratio based on standard uptake value (SUV) in a certain voxel normalized to mean SUV in the aorta; FMISO_TBR_ > 1.4 and FDG_TBR_ > 2.0, as defined previously but only taking into account voxels with TBR > 1.4 and 2.0, respectively.

## References

[1] Carey L. A., Perou C. M., Livasy C. A. (2006). Race, breast cancer subtypes, and survival in the carolina breast cancer study. *JAMA*.

[2] Wirapati P., Sotiriou C., Kunkel S. (2008). Meta-analysis of gene expression profiles in breast cancer: toward a unified understanding of breast cancer subtyping and prognosis signatures. *Breast Cancer Research*.

[3] Haynes B., Sarma A., Nangia-Makker P., Shekhar M. P. (2017). Breast cancer complexity: implications of intratumoral heterogeneity in clinical management. *Cancer and Metastasis Reviews*.

[4] Martelotto L. G., Ng C. K., Piscuoglio S., Weigelt B., Reis-Filho J. S. (2014). Breast cancer intra-tumor heterogeneity. *Breast Cancer Research*.

[5] Mann R. M., Balleyguier C., Baltzer P. A. (2015). Breast MRI: EUSOBI recommendations for women’s information. *European Radiology*.

[6] Pinker K., Bogner W., Baltzer P. (2014). Improved differentiation of benign and malignant breast tumors with multiparametric ^18^fluorodeoxyglucose positron emission tomography magnetic resonance imaging: a feasibility study. *Clinical Cancer Research*.

[7] Margolis N. E., Moy L., Sigmund E. E. (2016). Assessment of aggressiveness of breast cancer using simultaneous ^18^F-FDG-PET and DCE-MRI: preliminary observation. *Clinical Nuclear Medicine*.

[8] Cheng J., Lei L., Xu J. (2013). ^18^F-fluoromisonidazole PET/CT: a potential tool for predicting primary endocrine therapy resistance in breast cancer. *Journal of Nuclear Medicine*.

[9] Been L. B., Elsinga P. H., de Vries J. (2006). Positron emission tomography in patients with breast cancer using ^18^F-3′-deoxy-3′-fluoro-l-thymidine (^18^F-FLT)-a pilot study. *European Journal of Surgical Oncology (EJSO)*.

[10] Ruan K., Song G., Ouyang G. (2009). Role of hypoxia in the hallmarks of human cancer. *Journal of Cellular Biochemistry*.

[11] Okunieff P., Ding I., Vaupel P., Höckel M. (2003). Evidence for and against hypoxia as the primary cause of tumor aggressiveness. *Advances in Experimental Medicine and Biology*.

[12] Tatum J. L. (2006). Hypoxia: importance in tumor biology, noninvasive measurement by imaging, and value of its measurement in the management of cancer therapy. *International Journal of Radiation Biology*.

[13] Knäusl B., Hirtl A., Dobrozemsky G. (2012). PET based volume segmentation with emphasis on the iterative TrueX algorithm. *Zeitschrift für Medizinische Physik*.

[14] Rapisarda E., Bettinardi V., Thielemans K., Gilardi M. C. (2010). Image-based point spread function implementation in a fully 3D OSEM reconstruction algorithm for PET. *Physics in Medicine and Biology*.

[15] Solutions SM http://www.medical.siemens.com/siemens/en_GB/gg_nm_FBAs/files/brochures/Biograph/Biograph_spec_sheet_0507.pdf.

[16] Koh W. J., Rasey J. S., Evans M. L. (1992). Imaging of hypoxia in human tumors with [F-18]fluoromisonidazole. *International Journal of Radiation Oncology, Biology, Physics*.

[17] Zips D., Zöphel K., Abolmaali N. (2012). Exploratory prospective trial of hypoxia-specific PET imaging during radiochemotherapy in patients with locally advanced head-and-neck cancer. *Radiotherapy and Oncology*.

[18] Georg P., Andrzejewski P., Baltzer P. (2017). Changes in tumor biology during chemoradiation of cervix cancer assessed by multiparametric MRI and hypoxia PET. *Molecular Imaging and Biology*.

[19] Alduk A. M., Brcic I., Podolski P., Prutki M. (2016). Correlation of MRI features and pathohistological prognostic factors in invasive ductal breast carcinoma. *Acta Clinica Belgica*.

[20] Dietzel M., Baltzer P. A. T., Vag T. (2010). Application of breast MRI for prediction of lymph node metastases-systematic approach using 17 individual descriptors and a dedicated decision tree. *Acta Radiologica*.

[21] Kaiser C. G., Herold M., Krammer J. (2017). Prognostic value of “prepectoral edema” in MR-mammography. *Anticancer Research*.

[22] Wu J., Li B., Sun X. (2017). Heterogeneous enhancement patterns of tumor-adjacent parenchyma at MR imaging are associated with dysregulated signaling pathways and poor survival in breast cancer. *Radiology*.

[23] Bae M. S., Shin S. U., Ryu H. S. (2016). Pretreatment MR imaging features of triple-negative breast cancer: association with response to neoadjuvant chemotherapy and recurrence-free survival. *Radiology*.

[24] Kizildag Yirgin I., Arslan G., Ozturk E. (2016). Diffusion weighted MR imaging of breast and correlation of prognostic factors in breast cancer. *Balkan Medical Journal*.

[25] Durando M., Gennaro L., Cho G. Y. (2016). Quantitative apparent diffusion coefficient measurement obtained by 3.0Tesla MRI as a potential noninvasive marker of tumor aggressiveness in breast cancer. *European Journal of Radiology*.

[26] Rabasco P., Caivano R., Simeon V. (2017). Can diffusion-weighted imaging and related apparent diffusion coefficient be a prognostic value in women with breast cancer?. *Cancer Investigation*.

[27] Niikura N., Masuda S., Kumaki N. (2014). Prognostic significance of the Ki67 scoring categories in breast cancer subgroups. *Clinical Breast Cancer*.

[28] Yip C. H., Rhodes A. (2014). Estrogen and progesterone receptors in breast cancer. *Future Oncology*.

[29] Asano A., Ueda S., Kuji I. (2018). Intracellular hypoxia measured by F-^18^fluoromisonidazole positron emission tomography has prognostic impact in patients with estrogen receptor-positive breast cancer. *Breast Cancer Research*.

