# Supplementary material for: Sequential [18F]FDG-[18F]FMISO PET and Multiparametric MRI at 3T for Insights into Breast Cancer Heterogeneity and Correlation with Patient Outcomes: First Clinical Experience
Source: Contrast Media Mol Imaging. 2019 Jan 8;2019:1307247. doi: 10.1155/2019/1307247 (PMC6341235; doi:10.1155/2019/1307247)

**Supplementary Materials**

**Figure S1:** Example of the registration of the CT_FDG_, CT_FMISO_ and T_2w_ MRI: (A) CT_FDG_; (B) T_2w_ MRI; (C) fusion of registered A and B; (D) CT_FDG_; (E) CT_FMISO_; (F) fusion of registered D and E.


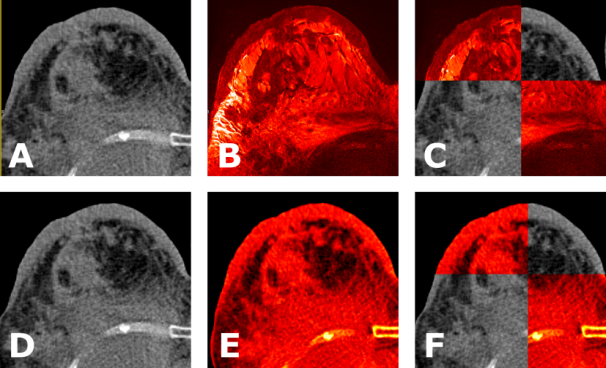


**Figure S2:** Graph showing numeric and color-coded Person’s correlation coefficients of all imaging parameters performed voxel-by-voxel within each patient’s delineated ROIs.

Abbreviations: T2w, T2 weighted magnetic resonance imaging (MRI); TIRM, turbo inversion recovery magnitude MRI; ADC, apparent diffusion coefficient; IE, initial enhancement ratio; WO wash out ratio; FMISO/FDG_TBR_, tumor to background ratio based on standard uptake value (SUV) in a certain voxel normalized to mean SUV in the aorta; FMISO_TBR_>1.4 and FDG_TBR_>2.0, as defined previously but only taking into account voxels with TBR > 1.4 and 2.0, respectively.


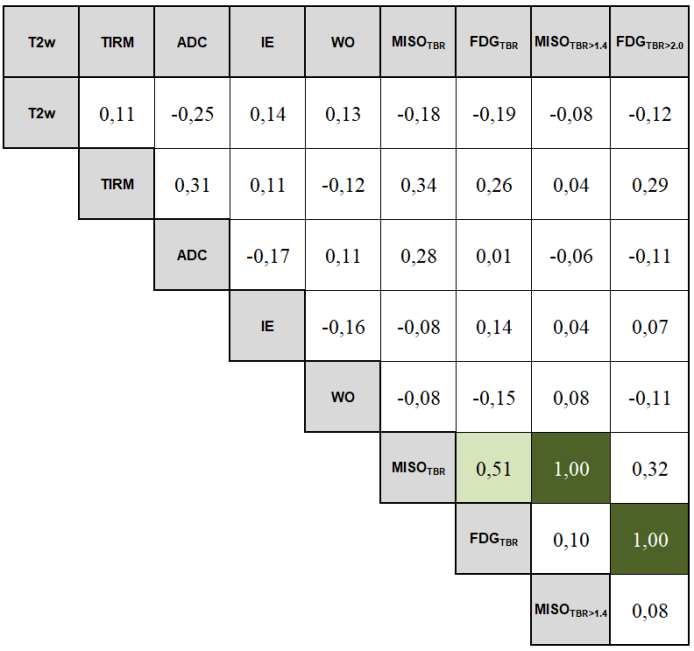

Supplement: Supplementary Materials — Figure S1: example of the registration of the CTFDG, CTFMISO, and T2w MRI: (A) CTFDG; (B) T2w MRI; (C) fusion of registered A and B; (D) CTFDG; (E) CTFMISO; (F) fusion of registered D and E. Figure S2: graph showing numeric and color-coded Person's correlation coefficients of all imaging parameters performed voxel-by-voxel within each patient's delineated ROIs. Abbreviations: T2w, T2-weighted magnetic resonance imaging (MRI); TIRM, turbo inversion recovery magnitude MRI; ADC, apparent diffusion coefficient; IE, initial enhancement ratio; WO washout ratio; FMISO/FDGTBR, tumor to background ratio based on standard uptake value (SUV) in a certain voxel normalized to mean SUV in the aorta; FMISOTBR > 1.4 and FDGTBR > 2.0, as defined previously but only taking into account voxels with TBR > 1.4 and 2.0, respectively. [file 1307247.f1.docx]
